# HMGB1 as a Key Mediator in Malignant Mesothelioma and a Potential Target for Asbestos-Related Cancer Therapy

**DOI:** 10.3390/toxics13060448

**Published:** 2025-05-28

**Authors:** Yi-Fang Zhong, Chan Ding, Chun-Ji Yao, Jia-Chun Wang, Min-Qian Feng, Xiao-Xue Gong, Lin Yu, Hua-Dong Xu, Hai-Ling Xia

**Affiliations:** School of Public Health, Hangzhou Medical College, Hangzhou 310013, China; sdsnt1911230304@163.com (Y.-F.Z.); 130232023252@hmc.edu.cn (C.D.); yaochunji@126.com (C.-J.Y.); jiachun_w1124@163.com (J.-C.W.); 130232024250@hmc.edu.cn (M.-Q.F.); xiaoxuegong@163.com (X.-X.G.); yulin091623@163.com (L.Y.)

**Keywords:** malignant mesothelioma, HMGB1, TLR4, asbestos-related tumors, tumor progression

## Abstract

Malignant mesothelioma (MM) is a highly aggressive cancer strongly associated with asbestos exposure, and accumulating evidence suggests that high mobility group box 1 (HMGB1) plays a central role in its pathogenesis. Our in vitro and in vivo experiments revealed that HMGB1 was highly expressed in MM. Both genetic and pharmacological inhibition of HMGB1 markedly suppressed MM cell viability, migration, and invasion, while inducing G1-phase cell cycle arrest and enhancing apoptosis. Interestingly, the inhibition of Toll-like receptor 4 (TLR4), achieved through both siRNA and TAK-242 treatment, not only suppressed tumor-promoting signals but also reduced HMGB1 expression, suggesting a self-amplifying HMGB1-TLR4 loop. Mechanistically, in vitro experiments indicated that suppression of HMGB1 and TLR4 was associated with decreased activation of NF-κB, AKT, and ERK pathways, which are involved in regulating MM cell survival and motility. In xenograft models, treatment with ethyl pyruvate (EP) and TAK-242 significantly suppressed tumor growth and HMGB1 expression, reinforcing their therapeutic potential. Given HMGB1’s influence on both tumor cell behavior and the immune microenvironment, targeting the HMGB1-TLR4 axis may not only provide a novel therapeutic strategy for MM but also offer insights into the mechanisms underlying asbestos-induced tumorigenesis, potentially guiding future prevention and intervention strategies in asbestos-exposed populations.

## 1. Introduction

Malignant mesothelioma (MM) is a highly aggressive neoplasm originating from mesothelial cells, strongly linked to occupational asbestos exposure [1]. Despite global efforts to curb asbestos use since the 1970s, the incidence and mortality of MM continue to rise—largely due to the long latency period of 20–40 years [2,3]. Analysis of the Global Burden of Disease (GBD) 2019 dataset from 1990 to 2019 showed that although age-standardized mortality and disability-adjusted life year (DALY) rates have declined, the absolute numbers of MM deaths and DALYs nearly doubled by 2019, with occupational asbestos exposure accounting for 91.7% of deaths and 85.2% of DALYs. Notably, the burden was particularly pronounced among individuals aged 75 and older, highlighting the enduring health consequences of asbestos exposure [4]. Moreover, the substantial burden of mesothelioma has influenced asbestos policy decisions, with the odds of a country prohibiting asbestos increasing 14.1-fold in the one to five years preceding a ban, and showing a 26% higher likelihood for each additional MM death per million people per year [5]. Even in regions such as China, where asbestos use has been banned, the mesothelioma burden continues to rise due to the disease’s long latency [6]. While the exposed populations are aging and the burden is expected to decline in the coming decades, emerging evidence suggested that mesothelioma cases in younger individuals were increasingly associated with genetic predispositions, such as germline BAP1 mutations, rather than direct asbestos exposure [7]. This highlights the potential persistence of the disease burden in susceptible populations.

Current diagnostic and therapeutic strategies for MM remain inadequate to significantly improve patient outcomes. Histopathological and immunohistochemical assessments, while considered the gold standards for MM diagnosis, are invasive and technically demanding, and often limited to symptomatic or advanced cases. Imaging and cytological methods, though less invasive, lack sufficient sensitivity and specificity for early-stage disease detection [8,9]. Thus, there is an urgent clinical need to develop reliable, minimally invasive, and highly sensitive biomarkers to facilitate early diagnosis and timely intervention. In parallel, the intrinsic resistance of MM to conventional chemotherapy and radiotherapy further underscores the necessity for innovative therapeutic strategies, such as personalized medicine and molecularly targeted therapies, to enhance treatment efficacy and improve survival rates [9,10].

Epidemiological evidence indicated that MM cases were linked to germline mutations of BAP1 and other cancer susceptibility genes [7]. These mutations enhance individual susceptibility to asbestos-induced carcinogenesis via gene–environment interactions (GxE) [11], with High Mobility Group Box 1 (HMGB1) playing a key mechanistic role [12]. Specifically, loss of BAP1 function reduces the deubiquitylation of HDAC1, which interacts with HMGB1 and BAP1 as a trimer, leading to hyperacetylation and nuclear release of HMGB1, thereby promoting mesothelioma progression [12]. HMGB1, a non-histone nuclear protein that regulates DNA repair and transcription, plays a critical role in tumorigenesis when released extracellularly as a damage-associated molecular pattern (DAMP) [13,14]. The pivotal role of HMGB1 in asbestos-induced carcinogenesis was first elucidated by researchers, who demonstrated that asbestos exposure induces HMGB1 release, triggering chronic inflammation and promoting mesothelioma development [15]. Recent work further refined this model, showing that HMGB1 released by mesothelial cells initiates carcinogenesis, while macrophage-derived HMGB1 sustains tumor growth and invasion at later stages [16]. Moreover, HMGB1 released to the cytoplasm promotes autophagy, a prosurvival process that enables some mesothelial cells to survive asbestos exposure and potentially undergo malignant transformation later [17]. Overexpression of HMGB1 has been observed in various malignancies, including gastrointestinal tumors [18], non-small cell lung cancer [19], and hepatocellular carcinoma [20], supporting its potential as a biomarker for early diagnosis and prognosis assessment. Studies have shown that MM patients exhibited significantly elevated serum HMGB1 levels compared to asbestos-exposed (AE) individuals and healthy controls [21,22], with hyperacetylated HMGB1 demonstrating high sensitivity and specificity for distinguishing MM from AE individuals [23]. Our previous findings further confirmed that serum HMGB1 levels were significantly higher in MM patients than in AE individuals, those with pleural plaques, and healthy controls [24,25]. Thus, HMGB1 fulfills essential biomarker criteria, including biological plausibility, clinical relevance, accessibility through non-invasive sampling, and measurable correlation with disease status, underscoring its value in MM diagnosis and prognosis.

Asbestos exposure remains the primary risk factor for MM, with approximately 80% of cases linked to occupational or environmental exposure [2]. Studies have shown that asbestos fibers stimulate both immune and MM cells to secrete acetylated HMGB1, which subsequently activates receptor-mediated signaling pathways and triggers inflammatory responses that promote tumor progression [17,26]. HMGB1 exerts its oncogenic effects mainly through interactions with the receptor for advanced glycation end-products (RAGE) and Toll-like receptor 4 (TLR4), activating downstream pathways which drive epithelial-mesenchymal transition (EMT), invasion, and metastasis [13]. Additionally, HMGB1 enhances extracellular matrix degradation by upregulating matrix metalloproteinases (MMPs), facilitated cell cycle progression via cyclin D1 and cyclin E, and promotes cytoskeletal remodeling to enhance cell motility [27]. Given its central role in MM pathogenesis, targeting HMGB1 and its associated signaling network represents a promising therapeutic strategy. In this study, we investigated the expression and functional roles of HMGB1 in MM both in vitro and in vivo, and evaluated the therapeutic potential of interfering with HMGB1-mediated signaling pathways.

## 2. Materials and Methods

### 2.1. Cell Culture and Treatments

Human MM cell lines MSTO-211H (CRL-2081) and NCI-H2452 (CRL-5946), along with normal mesothelial cell line MeT-5A (CRL-9444) cells, were purchased from American Type Culture Collection (ATCC). MSTO-211H and NCI-H2452 cells were cultured in RPMI-1640 medium (Cellmax, Beijing, China) supplemented with 10% fetal bovine serum (FBS, Corning, Steuben County, NY, USA, Origin in New Zealand), whereas MeT-5A cells were maintained in M199 medium (Cellmax, Beijing, China) containing 10% FBS. All cells were incubated at 37 °C in a humidified atmosphere with 5% CO_2_.

For gene silencing experiments, cells at 70–90% confluence in 6-well plates were transfected with 50 nM HMGB1- or TLR4-specific siRNA (sequences provided in Table S1) using Lipofectamine 3000 (Thermo Fisher Scientific, San Francisco, CA, USA) following the manufacturer’s protocol. After 48 h, RNA and protein expression levels were assessed, and functional assays were performed. Additionally, to further investigate the roles of HMGB1 and TLR4, cells were treated with 0, 2.5, 5, 10, or 20 mM ethyl pyruvate (EP; an HMGB1 inhibitor, Sigma-Aldrich, St. Louis, MO, USA) or 0, 25, 50, 100, or 200 µM TAK-242 (a selective TLR4 antagonist, Aladdin, Shanghai, China) for 6, 24, 48 or 72 h, respectively. Vehicle controls with equivalent DMSO concentrations were included. In selected experiments, inhibitor treatments were combined with siRNA transfection to evaluate potential synergistic effects on cell viability, migration, invasion, cell cycle distribution, and apoptosis.

### 2.2. RNA Extraction and Quantitative Real-Time PCR

Total RNA was extracted using TRIzol reagent (Thermo Fisher Scientific, USA) following the manufacturer’s instructions, and quantified using a NanoDrop 2000 spectrophotometer (Thermo Fisher Scientific, USA). Complementary DNA (cDNA) was synthesized using a Hifair^®^III 1st Strand cDNA synthesis SuperMix Kit (YEASEN, Shanghai, China). Quantitative real-time PCR (qRT-PCR) was performed using SYBR Green Master Mix (Takara, Japan) on a QuantStudio 7 Flex system (Applied Biosystems, Waltham, MA, USA). The reaction conditions were initial denaturation at 95 °C for 30 s, followed by 40 cycles of 95 °C for 3 s and 60 °C for 20 s, with a melting curve analysis at the end. GAPDH served as the internal control, and relative gene expression was calculated using the 2^−ΔΔCt^ method. Primer sequences were synthesized by Invitrogen (Table S1).

### 2.3. Cell Viability

Cells were seeded in 96-well plates at a density of 2.5 × 10^3^ cells/well. Following overnight adherence, cells were treated with EP or TAK-242 for 6, 24, 48, or 72 h. Cell viability was measured using the CCK-8 assay. The 10 µL of CCK-8 solution (Beyotime Institute of Biotechnology, Shanghai, China) was added to each well, incubated at 37 °C for 1 h, and absorbance was read at 450 nm (with 650 nm as reference).

### 2.4. Wound Healing Assay

Cells were seeded into 6-well plates at a density of 2 × 10^5^ cells/well and allowed to adhere overnight. Following attachment, the cells were treated with EP, TAK-242 or siRNA targeting HMGB1. When the cells reached approximately 90% confluency, three parallel linear scratch wounds were uniformly generated in each well. After washing with PBS, cells were cultured in medium containing 1% FBS. Wound closure was monitored and photographed at 0, 24, 48, and 72 h, and migration was quantified using ImageJ software (version 1.44).

### 2.5. Transwell Assay

For the migration assay, cells were pretreated with EP, TAK-242, or siRNA for 24 h. Following treatment, cells (1.6 × 10^4^ cells/well) were seeded into the upper chamber of a 24-well Transwell insert (Corning, USA) containing serum-free medium, while the lower chamber was filled with medium supplemented with 10% FBS as a chemoattractant. After 48 h of incubation, non-migrated cells on the upper surface of the membrane were carefully removed. The migrated cells on the lower surface were fixed, stained with 0.1% crystal violet, and quantified under a microscope. For the invasion assay, a similar protocol was applied using Matrigel-coated Transwell inserts (Corning, USA) to simulate the extracellular matrix barrier. After incubation for 36 h, the non-invaded cells and Matrigel were removed, and the invaded cells were fixed, stained, and counted.

### 2.6. Cell Cycle and Apoptosis

Cells were harvested and fixed in 70% ethanol at −20 °C overnight for cell cycle analysis. After washing with PBS, cells were stained with a cell cycle assay kit according to the manufacturer’s protocol and analyzed using a Muse Cell Analyzer (Merck, Boston, MA, USA). For apoptosis detection, cells were resuspended in culture medium containing 1% FBS, stained with a Annexin V & Dead Cell assay kit (Merck, USA) following the manufacturer’s instructions, and analyzed immediately. Detailed procedures have been described in previous studies [28,29].

### 2.7. Xenograft Mouse Model and Tissue Analysis

Female BALB/c nude mice (5–6 weeks old) were used to establish the MM xenograft model. MSTO-211H cells (5 × 10^6^ cells in 100 μL PBS) were subcutaneously injected into the flank of each mouse. When tumors reached approximately 50 mm^3^, mice were randomly divided into four groups (n = 6 per group): control (no treatment), model (PBS-treated), EP-treated (100 mg/kg), and TAK-242-treated (3 mg/kg). Treatments were administered intraperitoneally every two days for 6 weeks. Tumor dimensions were measured with calipers, and tumor volume was calculated using the formula: V = 0.5 × L × W^2^, where V represents tumor volume (mm^3^), L the longest diameter (mm), and W the perpendicular short diameter (mm). At the end of the study, mice were euthanized; tumors were excised, weighed, and processed for histological and immunohistochemical analysis. Tumor sections were stained with hematoxylin and eosin (H&E) for morphological evaluation, and immunohistochemistry (IHC) was performed to assess HMGB1 (Abcam, Shanghai, China) and TLR4 (Abcam, Shanghai, China) expression. Plasma HMGB1 levels were quantified using a Mouse HMGB1 ELISA kit (Wuhan Huamei Cusabio Biotech Co., Wuhan, China). The animal experiments were approved by the Experimental Animal Ethics Committee of Zhejiang Academy of Medical Sciences.

### 2.8. Statistical Analysis

Statistical analyses were performed using SPSS 29 (IBM, Armonk, NY, USA). Data are expressed as mean ± standard deviation (SD). Comparisons between two groups were conducted using an independent samples *t*-test, while differences among multiple groups were analyzed using a one-way ANOVA followed by Tukey’s post hoc test or Dunnett’s test. A *p*-value of <0.05 was considered statistically significant.

## 3. Results

### 3.1. HMGB1 Is Highly Expressed in MM Cells and Xenograft Tumors

**Figure 1:**
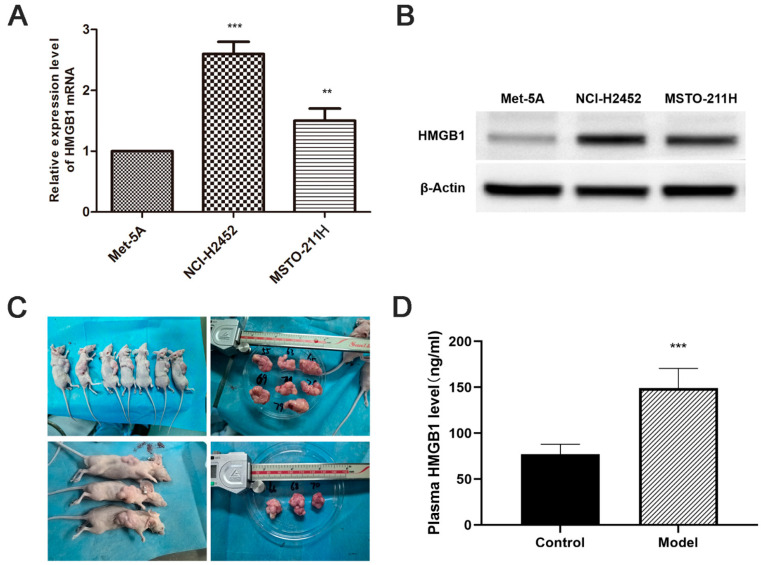
Elevated HMGB1 Expression in MM cell lines and xenograft models. (**A**) Relative HMGB1 mRNA levels in MM cell lines (NCI-H2452 and MSTO-211H) compared to normal mesothelial cells (MeT-5A), determined by quantitative real-time PCR. (**B**) Representative Western blot images showing HMGB1 protein expression in MM cell lines (NCI-H2452 and MSTO-211H) and normal mesothelial cells (MeT-5A). (**C**) Representative images of tumors from the MM xenograft model established by subcutaneous injection of MSTO-211H cells into BALB/c nude mice. (**D**) Plasma HMGB1 levels measured by ELISA in xenograft model mice. **, *p* < 0.01; ***, *p* < 0.001.

HMGB1 mRNA and protein levels were significantly upregulated in MM cell lines (NCI-H2452 and MSTO-211H) compared with normal mesothelial cells (MeT-5A) (Figure 1A,B). Consistent with the in vitro results, ELISA analysis showed a substantial increase in plasma HMGB1 levels in MM xenograft-bearing nude mice compared with normal controls (*p* < 0.05, Figure 1 C,D). These findings suggest that HMGB1 overexpression is a characteristic feature of MM in both in vitro and in vivo settings.

### 3.2. Interference with HMGB1 and TLR4 Reduces MM Cell Viability, Migration, and Invasion

To assess the functional role of HMGB1 and TLR4, we employed both siRNA-mediated knockdown and pharmacological inhibition. HMGB1 siRNA transfection significantly reduced HMGB1 expression in NCI-H2452 and MSTO-211H cells (Figure S1A–C). Similarly, EP treatment (5 and 10 mM) markedly decreased HMGB1 mRNA levels (Figure S1D,E). Additionally, TLR4 siRNA effectively downregulated TLR4 expression, accompanied by a significant reduction in HMGB1 mRNA levels (Figure S2A,B). TAK-242 treatment (100 and 200 μM) also significantly decreased both TLR4 and HMGB1 mRNA levels (Figure S2C,D).

**Figure 2:**
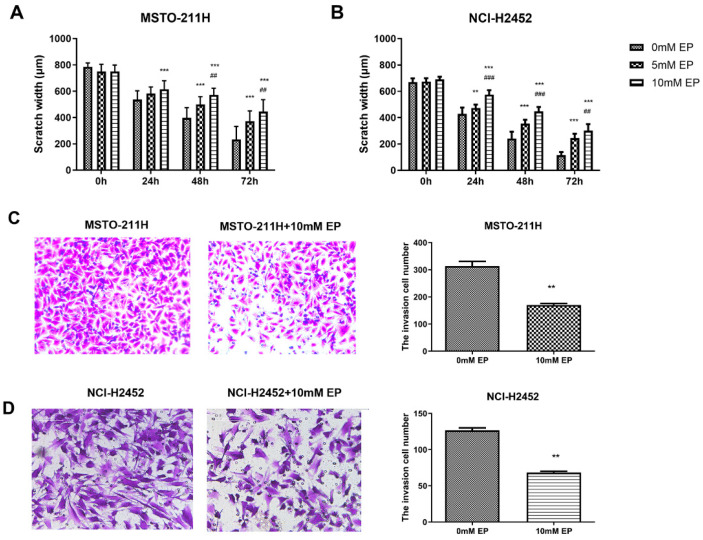
Effect of EP treatment on MM cell migration and invasion. Scratch wound healing assays showing the migration of MSTO-211H (**A**) and NCI-H2452 cells (**B**) treated with 0, 5, and 10 mM EP at 0, 24, 48, and 72 h. Representative images and quantification of the Transwell assays assessing invasion in MSTO-211H (**C**) and NCI-H2452 cells (**D**) treated with 0 and 10 mM EP. ** *p* < 0.01, *** *p* < 0.001 vs. 0 mM EP group; ## *p* < 0.01, ### *p* < 0.001 vs. 5 mM EP group.

**Figure 3:**
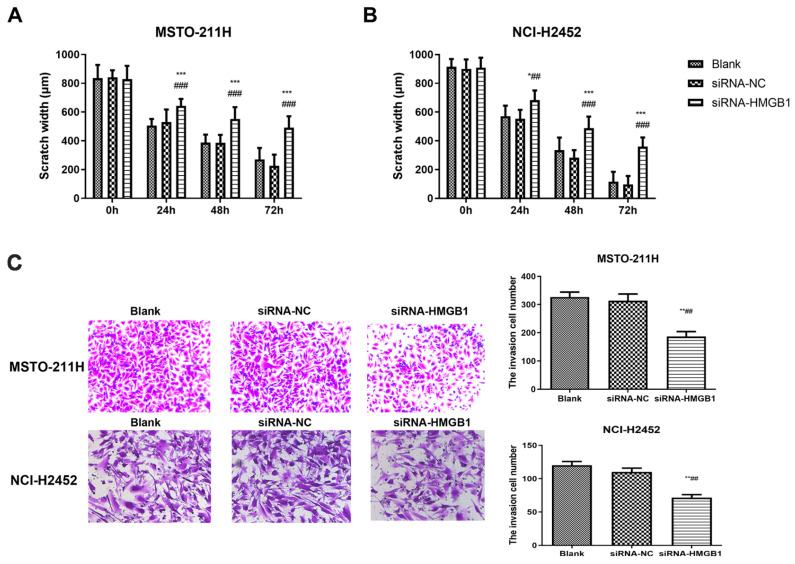
Effect of HMGB1 silencing on MM cell migration and invasion. Scratch wound healing assays showing the migration of MSTO-211H (**A**) and NCI-H2452 cells (**B**) treated with blank control, siRNA control, and HMGB1 siRNA at 0, 24, 48, and 72 h. Representative images and quantification of Transwell assays assessing invasion in MSTO-211H and NCI-H2452 cells (**C**) treated with blank control, siRNA control, and HMGB1 siRNA. * *p* < 0.05, ** *p* < 0.01, *** *p* < 0.001 vs. black group; ## *p* < 0.01, ### *p* < 0.001 vs. siRNA control group.

Following these interventions, EP and TAK-242 treatments led to dose- and time-dependent reductions in cell viability (Figure S3). Scratch wound healing and Transwell assays were used to evaluate the effects of HMGB1 inhibition on MM cell migration and invasion. EP treatment significantly suppressed the migration and invasion of NCI-H2452 and MSTO-211H cells. After EP treatment, the scratch closure rate was markedly reduced, and the number of cells passing through the Transwell chamber significantly decreased (Figure 2). Similarly, HMGB1 siRNA transfection significantly impaired cell migration and invasion compared with the control and siRNA-NC groups (Figure 3). Furthermore, TAK-242 treatment significantly reduced scratch closure rates and decreased the number of invasive cells in Transwell assays (Figure S4), further supporting the interplay between TLR4 and HMGB1 in regulating MM cell behavior.

### 3.3. HMGB1 Inhibition Alters Cell Cycle Progression and Induces Apoptosis

**Figure 4:**
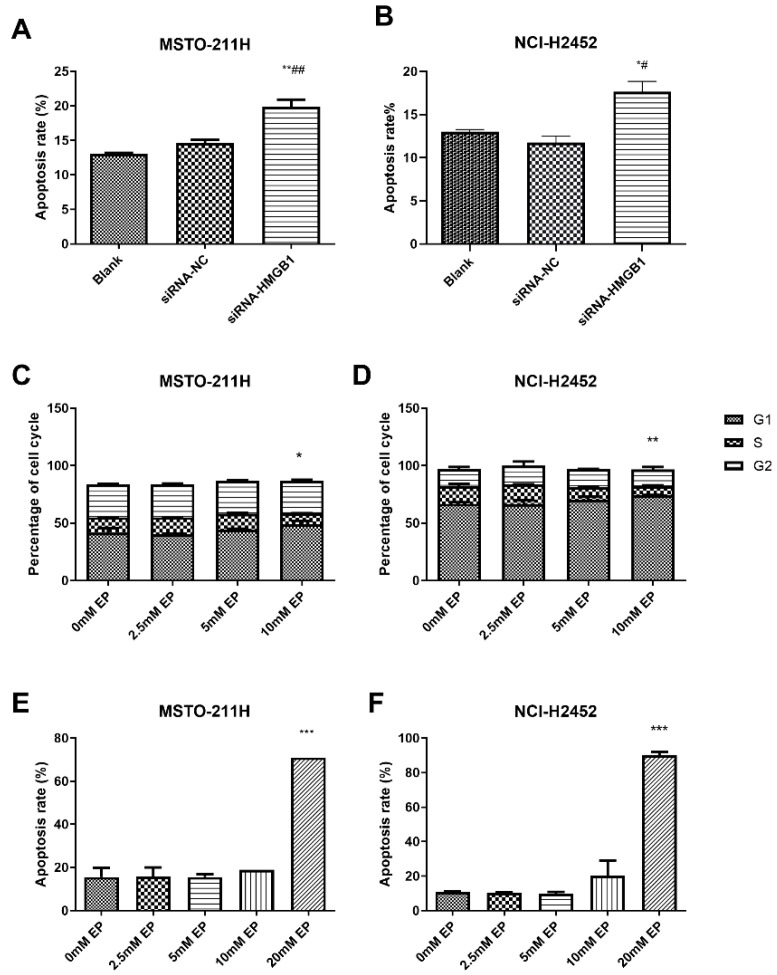
Effect of HMGB1 silencing and EP treatment on cell cycle arrest and apoptosis. Comparison of apoptosis in MSTO-211H (**A**) and NCI-H2452 (**B**) cells treated with blank control, siRNA control, and HMGB1 siRNA. Cell cycle distribution in MSTO-211H (**C**) and NCI-H2452 (**D**) cells treated with 0, 2.5, 5, and 10 mM EP. Apoptosis analysis of MSTO-211H (**E**) and NCI-H2452 (**F**) cells treated with 0, 2.5, 5, 10, and 20 mM EP. * *p* < 0.05, ** *p* < 0.01, *** *p* < 0.001 vs. control group; # *p* < 0.05, ## *p* < 0.01 vs. siRNA control group.

HMGB1 silencing significantly increased apoptosis in MSTO-211H and NCI-H2452 cells compared with the control and siRNA-NC groups (Figure 4A,B). EP treatment at 10 mM induced G1-phase arrest with a reduction in S-phase cells (Figure 4C,D), while apoptosis was significantly elevated at 20 mM EP (Figure 4E,F). Similarly, TLR4 inhibition via siRNA or TAK-242 reduced the S-phase population and increased apoptosis (Figure S5), with TLR4 silencing also downregulating HMGB1 expression (Figure S2), highlighting a regulatory link between TLR4 and HMGB1 in MM cell survival.

### 3.4. HMGB1-TLR4 Signaling Modulates Downstream Pathways

**Figure 5:**
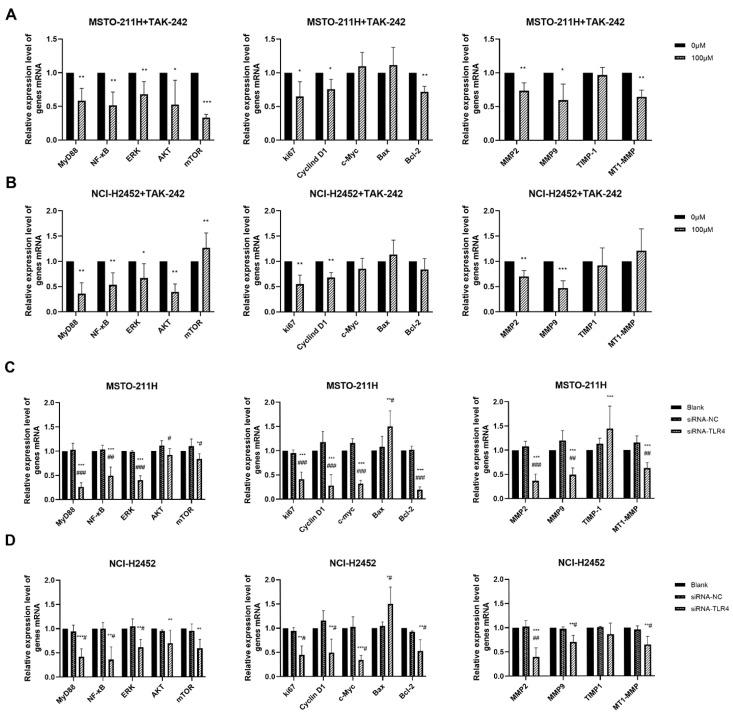
Effect of TAK-242 and TLR4 siRNA treatment on HMGB1-TLR4 signaling pathway in MM cells. The mRNA expression of key molecules in the HMGB1-TLR4 signaling axis in MSTO-211H (**A**) and NCI-H2452 (**B**) cells treated with 0 and 100 μM TAK-242. The mRNA expression of key molecules in the HMGB1-TLR4 signaling axis in MSTO-211H (**C**) and NCI-H2452 (**D**) cells treated with TLR4 siRNA. * *p* < 0.05, ** *p* < 0.01, *** *p* < 0.001 vs. control group; # *p* < 0.05, ## *p* < 0.01, ### *p* < 0.001 vs. siRNA control group.

To explore the molecular mechanisms underlying these functional changes, we examined the expression of key downstream molecules (Figure 5). TAK-242 and TLR4 siRNA treatment in MM cells not only reduced HMGB1 and TLR4 expression (Figure S3) but also significantly downregulated multiple key molecules, including MyD88, NF-κB, ERK, AKT, Ki67, Cyclin D1, MMP2/9, and MT1-MMP These results suggest that the HMGB1-TLR4 signaling axis regulates essential pathways involved in MM cell proliferation, invasion, and survival.

### 3.5. In Vivo Inhibition of HMGB1 Suppresses Tumor Growth

**Figure 6:**
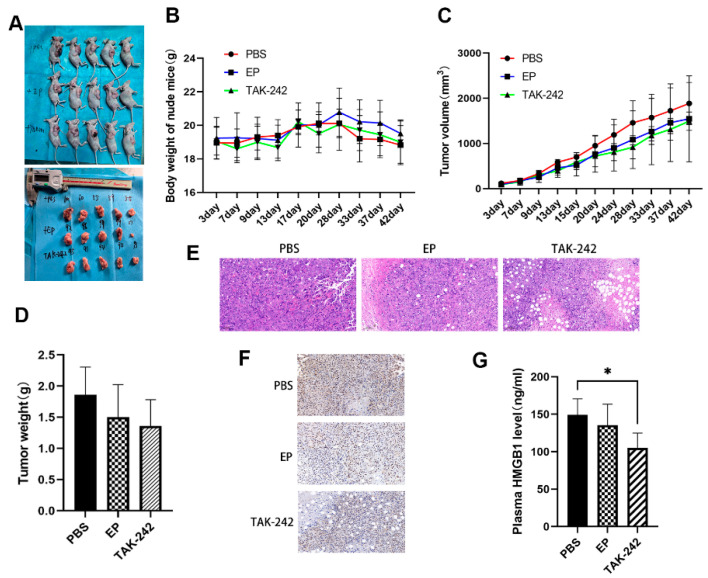
Effect of EP and TAK-242 treatment on MM xenograft model in vivo. (**A**) Representative images of mice and tumors after treatment treated with PBS, EP, or TAK-242. (**B**) Changes in body weight of mice over time after PBS, EP, or TAK-242 treatment. (**C**) Tumor volume measurements over time in mice treated with PBS, EP, or TAK-242. (**D**) Tumor weights in mice treated with PBS, EP, or TAK-242. (**E**) H&E staining of tumor tissues from PBS, EP, and TAK-242 treated groups. (**F**) Immunohistochemical staining showing HMGB1 expression in tumor tissues from the three treatment groups. (**G**) Plasma HMGB1 levels measured by ELISA in the three treatment groups. * *p* < 0.05.

In the MM xenograft model, nude mice treated with EP or TAK-242 exhibited slower tumor growth and lower tumor weights compared with the model group, although tumor volume differences were not always statistically significant (Figure 6A–D). Histological analysis of tumor tissues showed that EP and TAK-242 treatments resulted in decreased tumor cell density and increased necrosis (Figure 6E). Furthermore, immunohistochemical staining and ELISA demonstrated that both plasma and tumor tissue HMGB1 levels were significantly reduced in the TAK-242 treatment group (Figure 6F,G), underscoring the therapeutic potential of targeting the HMGB1-TLR4 axis in MM.

## 4. Discussion

Our study demonstrated that the HMGB1-TLR4 signaling axis played a pivotal role in the progression of MM and represented a promising target for therapeutic intervention. The in vitro data clearly indicated that interfering with HMGB1 via siRNA or inhibiting it with EP significantly reduced HMGB1 expression in MM cells, accompanied by corresponding reductions in migration, and invasion. These findings further established HMGB1 as a key driver of MM cell aggressiveness [30], and expanded on earlier research that first reported how EP suppressed the malignant phenotype of human mesothelioma by targeting HMGB1 [31]. In parallel, TLR4 inhibition, achieved either by siRNA or the selective antagonist TAK-242, further attenuated MM cell survival and motility and notably resulted in decreased HMGB1 levels. This suggested a potential positive feedback mechanism between HMGB1 and TLR4, where each reinforced the expression and activity of the other.

HMGB1 silencing and high-dose EP treatment induced G1-phase cell cycle arrest and increased apoptosis in MM cells. Recent studies have demonstrated that HMGB1 promotes cell cycle changes and is closely linked to chemoresistance by enhancing DNA damage repair and anti-apoptotic signaling [32,33]. Moreover, TLR4 signaling has been implicated in cell cycle regulation, with evidence suggesting its involvement in HMGB1-driven proliferative signaling [34]. Consistently, the observed decrease in S-phase cell populations following HMGB1 and TLR4 inhibition indicated that disruption of this axis interrupted critical proliferative signals, thereby sensitizing MM cells to apoptosis.

Mechanistically, our results demonstrated that the suppression of HMGB1 and TLR4 led to decreased activation of key downstream effectors, including NF-κB, AKT, and ERK. These molecules are well known regulators of cell survival, proliferation, and motility [13,35,36]. Their downregulation following targeted interventions suggested that the oncogenic effects of HMGB1 were mediated through these pathways. Specifically, inhibition of NF-κB reduced the transcription of anti-apoptotic genes [37], tipping the balance toward cell death. Similarly, reduced AKT and ERK activity likely contributed to diminished cell proliferation and impaired migratory capacity [38].

Our in vivo experiments further substantiated the therapeutic potential of targeting the HMGB1-TLR4 axis. In the xenograft mouse model, both EP and TAK-242 treatments resulted in significantly slower tumor growth and reduced tumor weight. These findings were consistent with prior research demonstrating that EP effectively reduced HMGB1-mediated tumor progression in other malignancies [39,40]. Additionally, TAK-242 has been shown to suppress tumor growth by modulating the tumor microenvironment and reducing pro-inflammatory cytokine production [41]. Importantly, these reductions in tumor burden were accompanied by decreased HMGB1 expression in both tumor tissues and plasma, further supporting the role of HMGB1 as a driver of MM aggressiveness. This in vivo evidence aligned with our in vitro findings and reinforced the therapeutic rationale for targeting HMGB1 signaling in MM.

A noteworthy aspect of our study was the demonstration that TLR4 inhibition not only reduced its own expression but also has a secondary effect on HMGB1 levels. Previous studies have reported similar self-amplifying loops between HMGB1 and TLR4 in other malignancies, where sustained activation of this axis enhances tumor aggressiveness [42,43]. Additionally, TLR4 inhibition has been shown to mitigate inflammatory cytokine production and tumor-promoting effects in colitis-associated colon cancer [44]. These findings support our hypothesis that the HMGB1-TLR4 axis may operate as a feed-forward loop, perpetuating tumor-promoting processes. Given that MM patients often exhibit persistently high HMGB1 levels despite reduced asbestos exposure, disrupting this loop may provide a dual benefit by simultaneously lowering both HMGB1 and TLR4 expression and activity.

Another important consideration is the role of HMGB1 in modulating the tumor microenvironment. While our study primarily focused on direct effects on MM cells, emerging evidence suggests that HMGB1 may also modulate the immune milieu [13,18,34]. Prior research has demonstrated that HMGB1 promotes the recruitment and expansion of immunosuppressive cells, such as myeloid-derived suppressor cells and regulatory T cells, contributing to immune evasion [45,46]. Notably, a previous study addressed the critical role of macrophages that release HMGB1 in the mesothelioma tissue microenvironment in promoting mesothelioma growth [16].Furthermore, extracellular HMGB1 interacts with pattern recognition receptors to sustain a pro-tumor inflammatory environment, enhancing resistance to immunotherapy [13,45,47]. Although we did not directly assess these immune parameters, the interplay between HMGB1-mediated signaling and immune regulation represents a promising avenue for future research, particularly in the context of combination therapies targeting both tumor cells and the tumor microenvironment.

Despite these promising findings, several limitations warrant discussion. First, while our in vitro models provided valuable insights into the cellular mechanisms underlying MM progression, they did not fully recapitulate the complexity of the tumor microenvironment. Although the xenograft mode offered more physiologically relevant system, its lacked of a functional immune system remains a limitation [48]. Future studies employing immunocompetent animal models could provide further insights into the interplay between HMGB1 signaling and host immune responses. Second, although our study demonstrated that targeting HMGB1 and TLR4 could inhibit key oncogenic processes in MM, the long-term effects and potential toxicities of such interventions remained to be elucidated in clinical settings. In this regard, safer HMGB1 inhibitors such as aspirin have been shown to effectively reduce asbestos-induced mesothelioma growth in mice [49], providing an alternative avenue for clinical translation.

## 5. Conclusions

This study highlighted the critical role of HMGB1 in the progression of MM, likely through its interaction with TLR4. Our results demonstrated that the HMGB1-TLR4 signaling axis was critically involved in the proliferation, migration, invasion, and survival of MM cells. Disrupting this axis, either through genetic or pharmacological approaches, significantly attenuated oncogenic behaviors both in vitro and in vivo. These findings reinforced the potential of HMGB1 as a diagnostic and prognostic biomarker and supported the development of targeted therapies aimed at this signaling pathway. Future research should focus on validating these findings in clinical trials and further investigating the role of HMGB1-TLR4 signaling in asbestos-induced MM, to better understand its implications in exposure-related tumorigenesis.

## Data Availability

The data presented in this study are available upon request from the corresponding author.

## Supplementary Materials

The following supporting information can be downloaded at: https://www.mdpi.com/article/10.3390/toxics13060448/s1, Table S1: Sequences of genes for qPCR and siRNA; Figure S1: Assessment of HMGB1 expression following HMGB1 siRNA knockdown and EP inhibition; Figure S2: Assessment of TLR4 and HMGB1 expression following TLR4 siRNA knockdown and TAK-242 treatment; Figure S3: Dose- and time-dependent effects of EP and TAK-242 treatments on cell viability of MM cells; Figure S4: Effect of TAK-242 treatment on MM cell migration and invasion; Figure S5: Effect of TLR4 inhibition on cell cycle and apoptosis in MM cells.
