# Supplementary material for: HMGB1 as a Key Mediator in Malignant Mesothelioma and a Potential Target for Asbestos-Related Cancer Therapy
Source: Toxics. 2025 May 28;13(6):448. doi: 10.3390/toxics13060448 (PMC12197314; doi:10.3390/toxics13060448)
Supplement: Supplementary file 1 [file toxics-13-00448-s001.zip › toxics-3622086-supplementary.pdf]

Table S1. Sequences of genes for qPCR and siRNA

Gene	Type	Sense (5’-3’)	Antisense (5’-3’)
HMGB1	siRNA	CCCGUUAUGAAAGAGAAAUTT	AUUUCUCUUUCAUAACGGGTT
TLR4	siRNA	CCUGCUGGAUGGUAAAUCATT	UGAUUUACCAUCCAGCAGGTT
Negative control	siRNA	UUCUCCGAACGUGUCACGUTT	ACGUGACACGUUCGGAGAATT
GAPDH	Primer for qPCR	ACGGATTTGGTCGTATTGGG	CTCGCTCCTGGAAGATGGTG
HMGB1	Primer for qPCR	GCGGACAAGGCCCGTTA	AGAGGAAGAAGGCCGAAGGA
TLR4	Primer for qPCR	GCCGAAAGGTGATTGTTGTGGTGT	TACCAGCACGACTGCTCAGAAACT
MyD88	Primer for qPCR	ACAGGCACCAGCATACAC	TTGGGTCCTTTCCAGAGT
NF-κB	Primer for qPCR	GGGGACTACGACCTGAATG	GGGCACGATTGTCAAAGAT
AKT	Primer for qPCR	CTGAGATTGTGTCAGCCCTGGA	CACAGCCCGAAGTCTGTGATCTTA
mTOR	Primer for qPCR	GCGCGGGCCCGGAGATGGTC	TGAAGCGCAGTAAGATTTTT
c-myc	Primer for qPCR	GCCACGTCTCCACACATCAG	TGGTGCATTTTCGGTTGTTG
ERK1	Primer for qPCR	AACTACCTACAGTCTCTGCCCTCT	GTGAGCCAGTGCTTCCTCTACT
ERK2	Primer for qPCR	TTGTCAGGACAAGGGCTCAGA	GGATAAAGCCAAGACGGGCTG
Bcl-2	Primer for qPCR	GCTTCAGGGTTTCATCCAG	GGCGGCAATCATCCTCTG

Bax	Primer for qPCR	GCCCTTTTGCTTCAGGGTTT;	TCCAATGTCCAGCCCATGAT
Ki67	Primer for qPCR	AAAGTGCCCAAGCCATAGA	CACCATTTGCCAGTTCCTC
Cyclin D1	Primer for qPCR	GATCAAGTGTGACCCGGACT	CTTGGGGTCCATGTTCT
TIMP-1	Primer for qPCR	AATTCCGACCTCGTCATCAG	TGCAGTTTTCCAGCAATGAG
MT1-MMP	Primer for qPCR	CCTTGGACTGTCAGGAATGAGG	TTCTCCGTGTCCATCCACTGGT
MMP2	Primer for qPCR	GAGAACCAAAGTCTGAAGAG	GGAGTGAGAATGCTGATTAG
MMP9	Primer for qPCR	CCTGGAGACCTGAGAACCAATC	CCACCCGAGTGTAACCATAGC

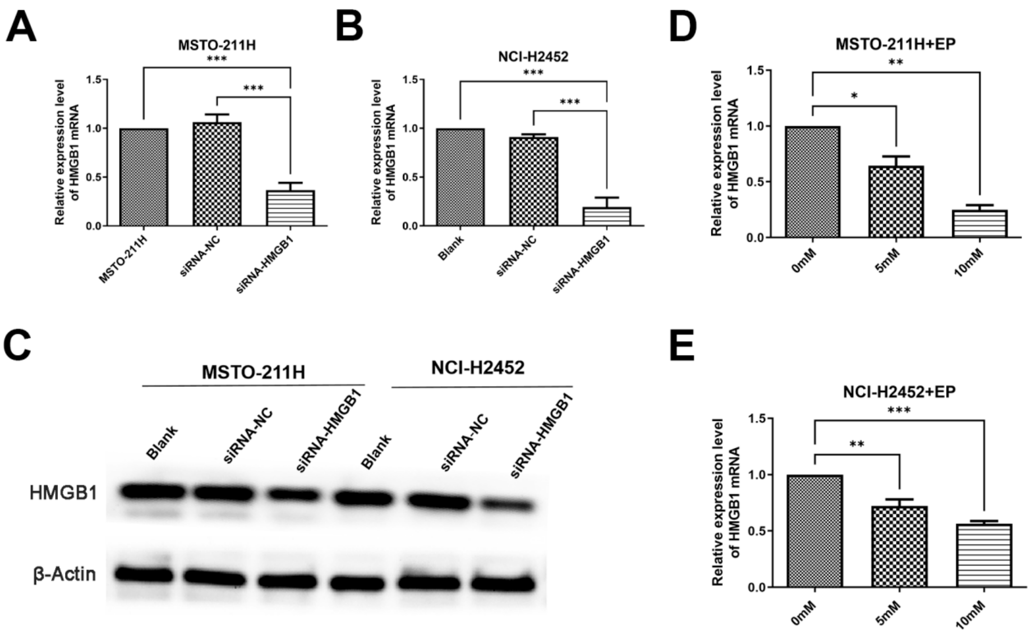

Figure S1. Assessment of HMGB1 expression following HMGB1 siRNA knockdown and EP inhibition. The mRNA expression of HMGB1 in MSTO-211H (A) and NCI-H2452 (B) cells treated with blank control, siRNA control, and HMGB1 siRNA. Representative Western blot images (C) showing HMGB1 expression in MSTO-211H and NCI-H2452 cells following treatment with blank control, siRNA control, and HMGB1 siRNA. The mRNA expression of HMGB1 in MSTO-211H (D) and NCI-H2452 (E) cells treated with 0, 5, and 10 mM EP. *$P < 0.05$, **$P < 0.01$, ***$P < 0.001$.

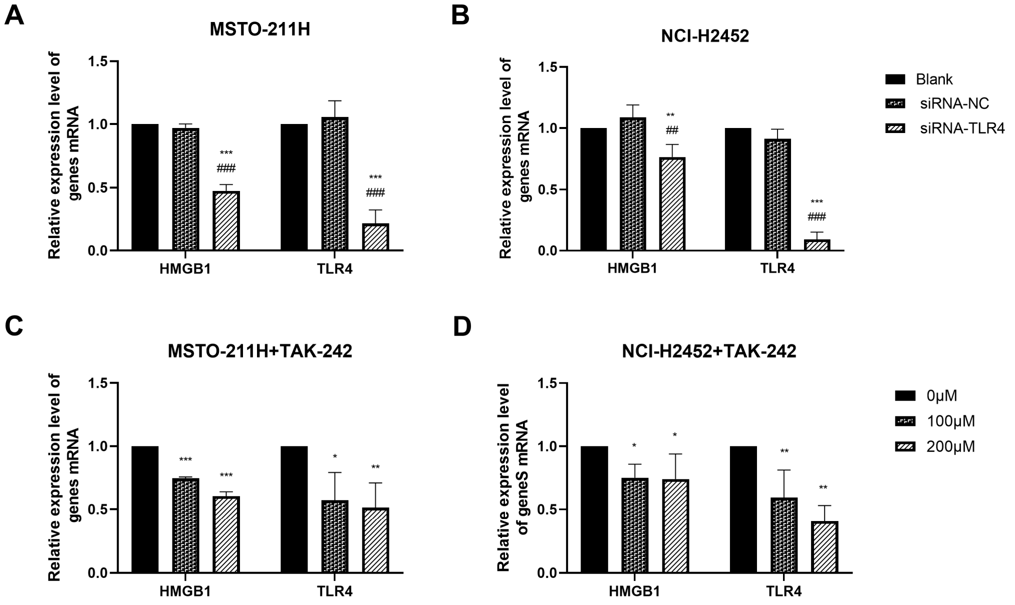

Figure S2. Assessment of TLR4 and HMGB1 expression following TLR4 siRNA knockdown and TAK-242 treatment. The mRNA expression of HMGB1 and TLR4 in MSTO-211H (A) and NCI-H2452 (B) cells treated with blank control, siRNA control, and TLR4 siRNA. The mRNA expression of HMGB1 and TLR4 in MSTO-211H (C) and NCI-H2452 (D) cells treated with 0, 100, and 200 μM TAK-242. *$P < 0.05$, **$P < 0.01$, ***$P < 0.001$ vs. control group; ##$P < 0.01$, ###$P < 0.001$ vs. siRNA control group.

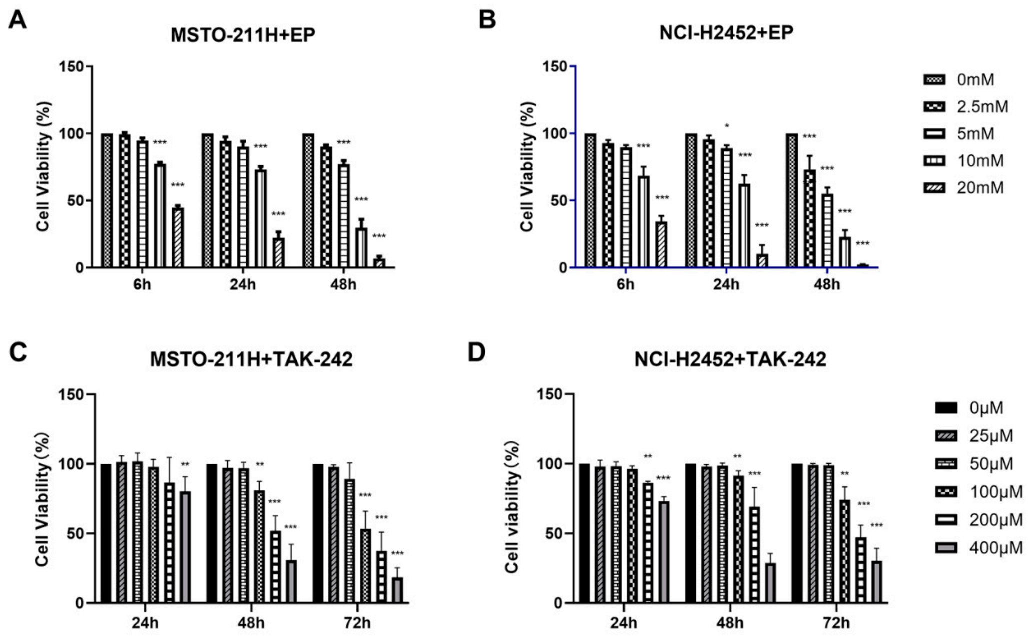

Figure S3. Dose- and time-dependent effects of EP and TAK-242 treatments on cell viability of MM cells. Cell viability of MSTO-211H (A) and NCI-H2452 (B) cells treated with increasing concentrations of EP (0, 2.5, 5, 10, and 20 mM) for 6, 24, and 48 hours. Cell viability of MSTO-211H cells (C) and NCI-H2452 (D) treated with increasing concentrations of TAK-242 (0, 25, 50, 100, 200, and 400 μM) for 24, 48, and 72 hours. *$P < 0.05$, **$P < 0.01$, ***$P < 0.001$.

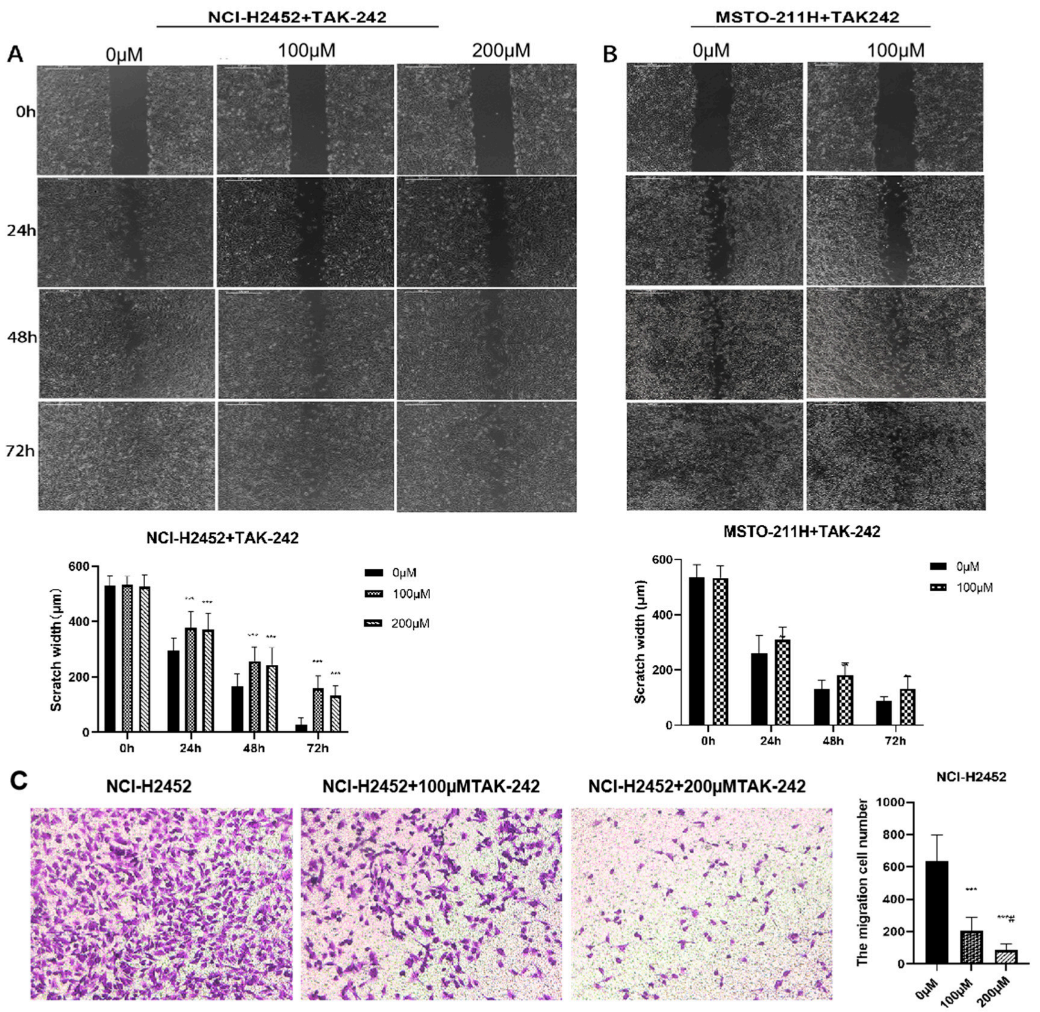

Figure S4. Effect of TAK-242 treatment on MM cell migration and invasion. Representative images and bar graph of the scratch wound healing assay for MSTO-211H cells **(A)** and NCI-H2452 (B) cells treated with TAK-242 for 0, 24, 48, and 72 h. **(C)** Representative images and bar graph of the Transwell assay for MSTO-211H cells treated with 0, 100, and 200 mM TAK-242. **$P < 0.01$, ***$P < 0.001$; #$P < 0.05$ vs. 100 µM group.

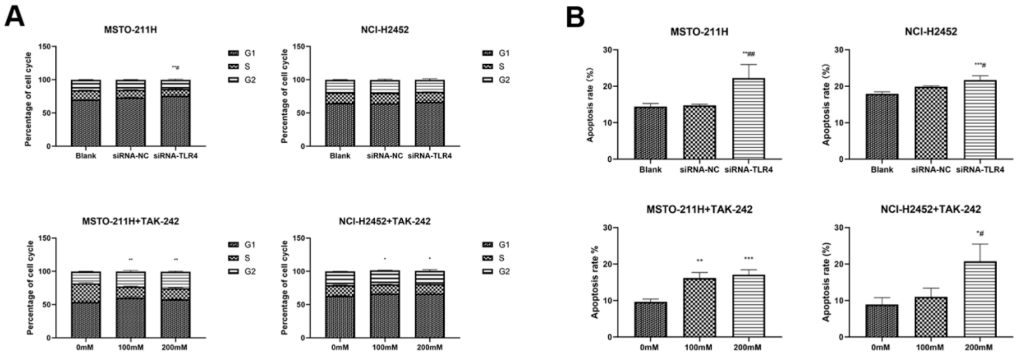

Figure S5. Effect of TLR4 inhibition on cell cycle and apoptosis in MM cells. (A) Cell cycle distribution in MSTO-211H and NCI-H2452 cells treated with siRNA or 0, 100, and 200 μM TAK-242 for 48 hours. (B) Apoptosis levels in MSTO-211H and NCI-H2452 cells treated with siRNA or 0, 100, and 200 μM TAK-242 for 48 hours. *$P < 0.05$, **$P < 0.01$, ***$P < 0.001$ vs. first group; #$P < 0.05$, ##$P < 0.01$ vs. middle group.
